# CNP-miR146a Promotes NRF2-Related Ferroptosis-Protective Myeloid Programs and Immune–Vascular Regeneration in Diabetic Wounds

**DOI:** 10.3390/pharmaceutics18091116

**Published:** 2026-09-04

**Authors:** Melisa Kafali, Emilia Mora Pinos, Katharina S. Berryman, Kellen Chen, Geoffrey C. Gurtner, Kenneth W. Liechty, Carlos Zgheib

**Affiliations:** 1 Laboratory for Fetal and Regenerative Biology, Department of Surgery, University of Arizona College of Medicine, Banner Children’s at Diamond Children’s Medical Center, Tucson, AZ 85721, USA; melisakafali@arizona.edu (M.K.); emiliamorapinos@arizona.edu (E.M.P.); kliechty@arizona.edu (K.W.L.); 2 Department of Surgery, University of Arizona College of Medicine, Tucson, AZ 85724, USA; katharinafischer@arizona.edu (K.S.B.); kellenchen@arizona.edu (K.C.); gurtner@surgery.arizona.edu (G.C.G.); 3 Ceria Therapeutics, Inc., Tucson, AZ 85721, USA

**Keywords:** diabetic wound healing, ferroptosis, NRF2, cerium oxide nanoparticles, microRNA-146a, single-cell RNA sequencing, angiogenesis

## Abstract

**Background:** Diabetic wounds are characterized by chronic inflammation, oxidative stress, impaired angiogenesis, and delayed tissue repair. Ferroptosis has emerged as a potential contributor to diabetic wound pathology; however, its relationship with impaired tissue regeneration remains incompletely understood. **Objectives:** We investigated cellular and transcriptional responses associated with cerium oxide nanoparticle-conjugated microRNA-146a (CNP-miR146a) treatment and whether wound repair is associated with ferroptosis-protective programs and immune–vascular communication. **Methods:** Diabetic excisional wounds were treated with CNP-miR146a or phosphate-buffered saline controls. Single-cell RNA sequencing was performed on wound tissues, with primary analyses focused on postoperative Day 7. Cellular composition, ferroptosis-associated programs, pseudotime trajectories, and inferred ligand–receptor communication networks were analyzed. **Results:** CNP-miR146a treatment was associated with transcriptional remodeling of the diabetic wound microenvironment, with myeloid cells exhibiting a prominent response. Treatment was associated with higher NRF2-related antioxidant, ferroptosis-protective, and iron-homeostasis transcriptional programs and with a repair-associated myeloid state. Pseudotime analysis identified a trajectory from monocytes toward pro-regenerative macrophages accompanied by dynamic expression of antioxidant, iron-homeostasis, and repair-associated genes. CellChat predicted increased immune–vascular communication through angiogenic and extracellular matrix-associated pathways. Endothelial cells exhibited increased NRF2-associated transcriptional programs, angiogenesis-associated gene expression, and endothelial repair markers. **Conclusions:** CNP-miR146a-mediated wound repair is associated with coordinated ferroptosis-protective and NRF2-related transcriptional programs, pro-regenerative myeloid states, endothelial angiogenesis-associated programs, and predicted immune–vascular communication. These findings identify ferroptosis-associated and immune–vascular transcriptional networks as candidate mechanisms of CNP-miR146a-mediated diabetic wound repair requiring further functional validation.

## 1. Introduction

Diabetes mellitus affects more than 500 million individuals worldwide and represents a major public health challenge [1,2]. The economic burden of diabetes is substantial, accounting for an estimated $412.9 billion in healthcare expenditures in the United States in 2022 [1,3]. Among its complications, diabetic foot ulcers are particularly devastating, affecting approximately one-third of patients during their lifetime [4] and frequently leading to infection, hospitalization, lower-extremity amputation, and increased mortality [4,5,6]. Despite advances in wound care, many diabetic wounds remain chronically non-healing due to persistent inflammation, excessive oxidative stress, impaired angiogenesis, and dysregulated tissue repair [7]. Consequently, there remains a critical need to identify the molecular mechanisms that impair wound healing and to develop therapies capable of restoring regenerative tissue repair.

Successful wound healing requires coordinated interactions among immune cells, endothelial cells, fibroblasts, and keratinocytes [4]. Following injury, inflammatory cells remove damaged tissue and pathogens, endothelial cells initiate neovascularization to restore oxygen and nutrient delivery, and fibroblasts remodel extracellular matrix to support tissue regeneration [8]. In diabetes, however, chronic inflammation and oxidative stress disrupt these tightly regulated processes, resulting in impaired angiogenesis, defective tissue remodeling, and delayed wound closure. Increasing evidence suggests that altered communication between immune and vascular compartments plays a central role in the pathogenesis of chronic diabetic wounds [4,9].

Ferroptosis has recently emerged as an important contributor to diabetic wound pathology. Recent studies have implicated ferroptosis in a variety of diabetic complications, including nephropathy, retinopathy, cardiomyopathy, and impaired wound healing [10,11]. Ferroptosis is an iron-dependent form of regulated cell death characterized by excessive lipid peroxidation, oxidative stress, and failure of cellular antioxidant defense systems [12]. Persistent hyperglycemia promotes mitochondrial dysfunction, reactive oxygen species accumulation, chronic inflammation, and disturbances in iron metabolism that collectively favor ferroptotic injury [13,14]. Within diabetic wounds, both immune and endothelial cell populations appear particularly susceptible to ferroptosis-associated stress, which may contribute to persistent inflammation, endothelial dysfunction, and defective tissue regeneration [10,15].

Cellular resistance to ferroptosis is largely regulated by antioxidant and iron-buffering pathways controlled by nuclear factor erythroid 2-related factor 2 (NRF2) [16]. As a master regulator of cellular redox homeostasis, NRF2 coordinates transcriptional programs involved in glutathione metabolism, reactive oxygen species detoxification, iron sequestration, and cellular stress adaptation [16,17]. Activation of NRF2-dependent pathways promotes cellular resilience to oxidative stress and has emerged as a promising therapeutic strategy for diabetic wound repair [18]. However, the cell-type-specific mechanisms through which ferroptosis influences tissue regeneration remain incompletely understood.

Recent evidence suggests that ferroptosis may affect wound healing not only through regulation of cell survival but also through modulation of intercellular communication networks [19]. Myeloid cells regulate angiogenesis through secretion of cytokines, growth factors, and extracellular matrix-remodeling molecules that influence endothelial cell survival, migration, and vessel formation [20]. Conversely, endothelial cells actively regulate immune-cell recruitment and activation through chemokines, cytokines, and adhesion molecules. Disruption of this immune–vascular crosstalk contributes to chronic inflammation, impaired angiogenesis, and defective tissue repair [21]. While signaling pathways such as vascular endothelial growth factor (VEGF), oncostatin M (OSM), and thrombospondin (THBS) have been implicated in wound healing, it remains unclear whether ferroptosis-associated dysfunction within myeloid cells contributes to impaired communication with endothelial cells during diabetic wound repair [22,23,24].

Cerium oxide nanoparticles (CNPs) possess unique catalytic antioxidant properties that enable repeated scavenging of reactive oxygen species and modulation of cellular redox signaling [25]. Previous studies have demonstrated that CNPs reduce oxidative stress, activate cytoprotective pathways, and enhance tissue repair in multiple preclinical models [25,26,27,28]. Importantly, CNPs have been shown to regulate NRF2-dependent antioxidant signaling and suppress ferroptosis-associated cellular injury [29]. MicroRNA-146a (miR-146a) is a key regulator of inflammation that suppresses NF-κB signaling through inhibition of IRAK1 and TRAF6 [30,31,32]. Reduced miR-146a expression has been observed in diabetic wounds and contributes to persistent inflammatory activation and impaired healing. Restoration of miR-146a expression reduces inflammation and improves wound repair [32].

Our previous studies demonstrated that conjugation of miR-146a to cerium oxide nanoparticles (CNP-miR146a) combines antioxidant and anti-inflammatory activities to accelerate diabetic wound healing [25,33,34]. Although these studies established the therapeutic efficacy of CNP-miR146a, the cellular mechanisms responsible for treatment-induced tissue regeneration remain poorly defined. In particular, it remains unclear whether CNP-miR146a-mediated wound repair is associated with ferroptosis-protective transcriptional programs within specific cellular populations and alterations in regenerative communication between immune and vascular compartments.

In the present study, we employed single-cell RNA sequencing, pseudotime trajectory analysis, and ligand–receptor interaction modeling to define the cellular mechanisms underlying CNP-miR146a-mediated diabetic wound repair. We hypothesized that CNP-miR146a-mediated wound repair is associated with NRF2-related, ferroptosis-protective myeloid transcriptional programs, endothelial regenerative responses, and alterations in immune–vascular communication.

## 2. Materials and Methods

### 2.1. Synthesis and Characterization of CNP-miR146a

Cerium oxide nanoparticles (CNPs) were synthesized using a previously published wet-chemistry method [25]. Briefly, cerium nitrate hexahydrate (99.999% purity; Sigma-Aldrich, St. Louis, MO, USA) was dissolved in ultrapure water and mixed thoroughly. Hydrogen peroxide (H_2_O_2_; Sigma-Aldrich, St. Louis, MO, USA) was added as an oxidizing agent while maintaining the reaction pH below 4, resulting in the formation of ultra-small crystalline CNPs. Surface conjugation of microRNA-146a (miR-146a) was performed using 1,1-carbonyldiimidazole (CDI) chemistry as previously described [35]. In this reaction, CDI generates a reactive intermediate that facilitates covalent bond formation between the free amine group located at the 3′ end of miR-146a and hydroxyl groups present on the nanoparticle surface.

Following conjugation, CNP-miR146a was dialyzed against RNase/DNase-free water and stored at −20 °C until use. Nanoparticle size and surface charge were characterized using a Zetasizer Nano instrument (Malvern Instruments, Malvern, UK).

Unconjugated CNPs exhibited a hydrodynamic diameter of approximately 20 nm and a zeta potential of +27 mV. Following miR-146a conjugation, the hydrodynamic size increased to approximately 190 nm and the zeta potential shifted to −18 mV, consistent with this surface modification [34]. Transmission electron microscopy characterization reported primary particle sizes of approximately 3–5 nm for unconjugated CNPs and 3–7 nm following miR-146a conjugation [34].

A quantitative polydispersity index (PDI) was not reported for the CNP-miR146a formulation used in this study and is therefore not available for retrospective reporting. Successful conjugation was supported by the previously characterized changes in hydrodynamic size and surface charge; however, quantitative miR-146a conjugation/loading efficiency, defined as the percentage of input miR-146a associated with the CNPs, was not determined for this formulation. Accordingly, no quantitative conjugation efficiency value is reported.

### 2.2. Diabetic Murine Wound Healing Model

All animal procedures were approved by the Institutional Animal Care and Use Committee (IACUC, protocol number: 2022-0934) of the University of Arizona and performed in accordance with institutional guidelines.

Twelve-week-old female diabetic db/db mice carrying the Leprdb mutation and age-matched heterozygous non-diabetic littermates (db/+) (BKS.Cg-Dock7m+/+Leprdb/J; Jackson Laboratory, Bar Harbor, ME, USA) were used. These 15 mice were assigned to three experimental groups (Het PBS, *n* = 5; Hom PBS, *n* = 5; Hom T, *n* = 5). Animals were anesthetized with inhaled isoflurane, and the dorsal skin was shaved, depilated, and sterilized with alcohol and povidone-iodine solution. Extended-release buprenorphine (Ethiqa XR^®^, Fidelis Animal Health, Inc., North Brunswick, NJ, USA; 0.05 mL per mouse) was administered subcutaneously for perioperative analgesia.

A single full-thickness excisional wound, including the panniculus carnosus, was created on the dorsum using an 8 mm biopsy punch (Med Vet International, Mettawa, IL, USA). Mice were assigned to three experimental groups: heterozygous PBS-treated controls (Het PBS), diabetic PBS-treated controls (Hom PBS), and diabetic mice treated with CNP-miR146a (Hom T).

The present scRNA-seq study was designed to compare the previously established CNP-miR146a formulation with PBS-treated diabetic and non-diabetic controls. CNP-only and free miR-146a treatment groups were not included in the current scRNA-seq experiment; however, the individual components and the conjugated formulation have been evaluated previously in the diabetic wound model [35].

At the time of injury, 100 ng of CNP-miR146a conjugate in 50 μL of PBS per wound was administered intradermally as five separate 10 μL injections, whereas control wounds received an equivalent volume of PBS (Gibco, Thermo Fisher Scientific, Waltham, MA, USA). Four injections were delivered at the wound margins corresponding to the 12, 3, 6, and 9 o’clock positions, while a fifth injection was placed at the wound center to ensure uniform distribution. Wounds were covered with a transparent occlusive dressing (Tegaderm^®^, 3M, St. Paul, MN, USA). Wound tissues were collected on postoperative Days 3 and 7 for single-cell RNA sequencing. The primary mechanistic analyses presented in this study focused on Day 7 samples, whereas Day 3 data were analyzed separately to provide additional temporal context and are presented in Supplementary Figure S1.

### 2.3. Preparation of Single-Cell Suspensions

Excised wound tissues were immediately placed in ice-cold Hanks’ Balanced Salt Solution (HBSS; Sigma-Aldrich, St. Louis, MO, USA) and mechanically minced into small fragments. Tissue digestion was performed using 0.25 mg/mL Liberase (Roche Diagnostics, Mannheim, Germany) solution for approximately 50 min with five intermittent pipetting steps.

Digestion was terminated by addition of Dulbecco’s Modified Eagle Medium (DMEM; Thermo Fisher Scientific, Waltham, MA, USA) supplemented with 10% fetal bovine serum (FBS; Thermo Fisher Scientific, Waltham, MA, USA). Cell suspensions were sequentially filtered through 100 μm and 70 μm cell strainers (Corning Inc., Tewksbury, MA, USA) to remove debris and undigested tissue fragments.

Following filtration, cells were centrifuged at 300× *g* for 8 min at 4 °C and resuspended in DMEM. Cell viability and concentration were assessed prior to library preparation. Samples used for single-cell RNA sequencing exhibited viability greater than 80% and were adjusted to approximately 1200 cells/μL.

### 2.4. Single-Cell RNA Sequencing and Library Preparation

Single-cell RNA sequencing libraries were generated using the Chromium Single Cell 3′ Gene Expression platform (10x Genomics, Pleasanton, CA, USA) according to the manufacturer’s instructions. Cell capture, barcoding, reverse transcription, cDNA amplification, and library preparation were performed by the University of Arizona Genetics Core.

Library quality was assessed using Fragment Analyzer-based quality control, and sequencing was performed using an AVITI platform (Element Biosciences, San Diego, CA, USA) with paired-end 2 × 75 bp chemistry.

Raw sequencing data were processed using Cell Ranger (10x Genomics) for demultiplexing, alignment to the mouse reference genome (mm10), and generation of gene-barcode matrices.

### 2.5. Single-Cell Transcriptomic Analysis

Downstream single-cell RNA-sequencing analyses were performed in R (version 4.6.0) using Seurat v5.5. Cells were retained after quality-control filtering if they contained >500 and <4000 detected genes and <10% mitochondrial transcripts. No minimum UMI-count threshold was applied. Cells with >4000 detected genes per cell were excluded as part of the doublet/high-complexity cell filtering criterion. Following quality-control filtering, data were normalized, integrated across experimental groups, scaled, and subjected to dimensionality reduction and unsupervised clustering using the Seurat workflow.

Uniform Manifold Approximation and Projection (UMAP) was used for visualization of the transcriptional landscape. Major cellular populations were annotated using canonical marker genes and classified as myeloid, fibroblast, endothelial, or lymphoid cells. Following QC, the postoperative Day 7 dataset contained 19,088 cells from Het PBS, 1382 cells from Hom PBS, and 9027 cells from Hom T, yielding 29,497 cells for downstream Day 7 analyses. For the Day 7 Hom T condition, the initial sample yielded 580 cells after QC. Due to the low cell yield, the experimental group was repeated and a second sample was submitted for 10x scRNA-seq, yielding 8447 cells after QC. The two Day 7 Hom T datasets were processed using the same quality-control criteria and were subsequently combined during the Seurat integration workflow prior to downstream analysis. Integration was performed together with the other experimental datasets to reduce technical variation across sequencing runs while preserving biological transcriptional differences. Cell numbers before and after quality-control filtering for each scRNA-seq dataset are provided in Table S1.

Although scRNA-seq datasets were generated at postoperative Days 3 and 7, the primary mechanistic analyses in the present study focused on postoperative Day 7 samples from Het PBS, Hom PBS, and Hom T groups. Day 3 data were analyzed separately to provide additional temporal context and are presented in Supplementary Figure S1. Differential expression analyses were performed to characterize treatment-associated transcriptional changes. Ferroptosis-related, iron-homeostatic, and NRF2-associated transcriptional programs were quantified using Seurat AddModuleScore with predefined gene sets listed in Supplementary Table S2. Sensitivity analyses using reduced core gene sets were additionally performed to evaluate the robustness of the observed transcriptional patterns to gene-set composition (Supplementary Figure S2 and Table S3).

Myeloid cell-state trajectories were inferred using Slingshot (version 2.20.0) to evaluate transcriptional progression among identified myeloid populations. Cell–cell communication networks were inferred using CellChat (version 2.2.0.9001) from normalized RNA expression data and broad cell-type annotations. CellChat analysis was performed separately for each experimental condition using the mouse ligand–receptor database (CellChatDB.mouse). Following database assignment, signaling-relevant genes were retained using subsetData(), and overexpressed genes and ligand–receptor interactions were identified using identifyOverExpressedGenes() and identifyOverExpressedInteractions(), respectively. Communication probabilities were inferred using the triMean method (computeCommunProb, type = “triMean”), and interactions involving cell populations containing fewer than 10 cells were excluded using filterCommunication(min.cells = 10). Pathway-level communication probabilities were subsequently calculated using computeCommunProbPathway(), and signaling networks were aggregated using aggregateNet(). CellChat objects generated for individual conditions were subsequently merged for comparative analyses. Comparisons between untreated diabetic (Hom PBS) and CNP-miR146a-treated diabetic (Hom T) wounds included pathway-level information flow and inferred myeloid–endothelial ligand–receptor communication. CellChat-derived interactions were interpreted as predicted transcriptomic communication networks rather than experimentally validated molecular interactions.

### 2.6. Statistical Analysis

Wound tissues from five animals within each experimental group were pooled prior to tissue dissociation and generation of the single-cell suspension. Consequently, the resulting scRNA-seq datasets represent pooled group-level profiles and do not contain independent animal-level biological replicates. Individual cells were therefore not considered independent biological replicates for treatment-level statistical inference.

Cell-type proportions were summarized descriptively across Het PBS, Hom PBS, and Hom T groups. Module score and normalized gene expression distributions were visualized at the single-cell level using violin plots to characterize treatment-associated transcriptional patterns. Because independent animal-level scRNA-seq replicates were not available, inferential statistical testing of treatment effects on cell-type proportions, module scores, and gene-expression distributions was not performed. Accordingly, differences among experimental groups are interpreted as descriptive and exploratory transcriptional patterns rather than animal-level statistical effects.

## 3. Results

### 3.1. Cellular Composition of Diabetic Wounds Following CNP-miR146a Treatment

**Figure 1:**
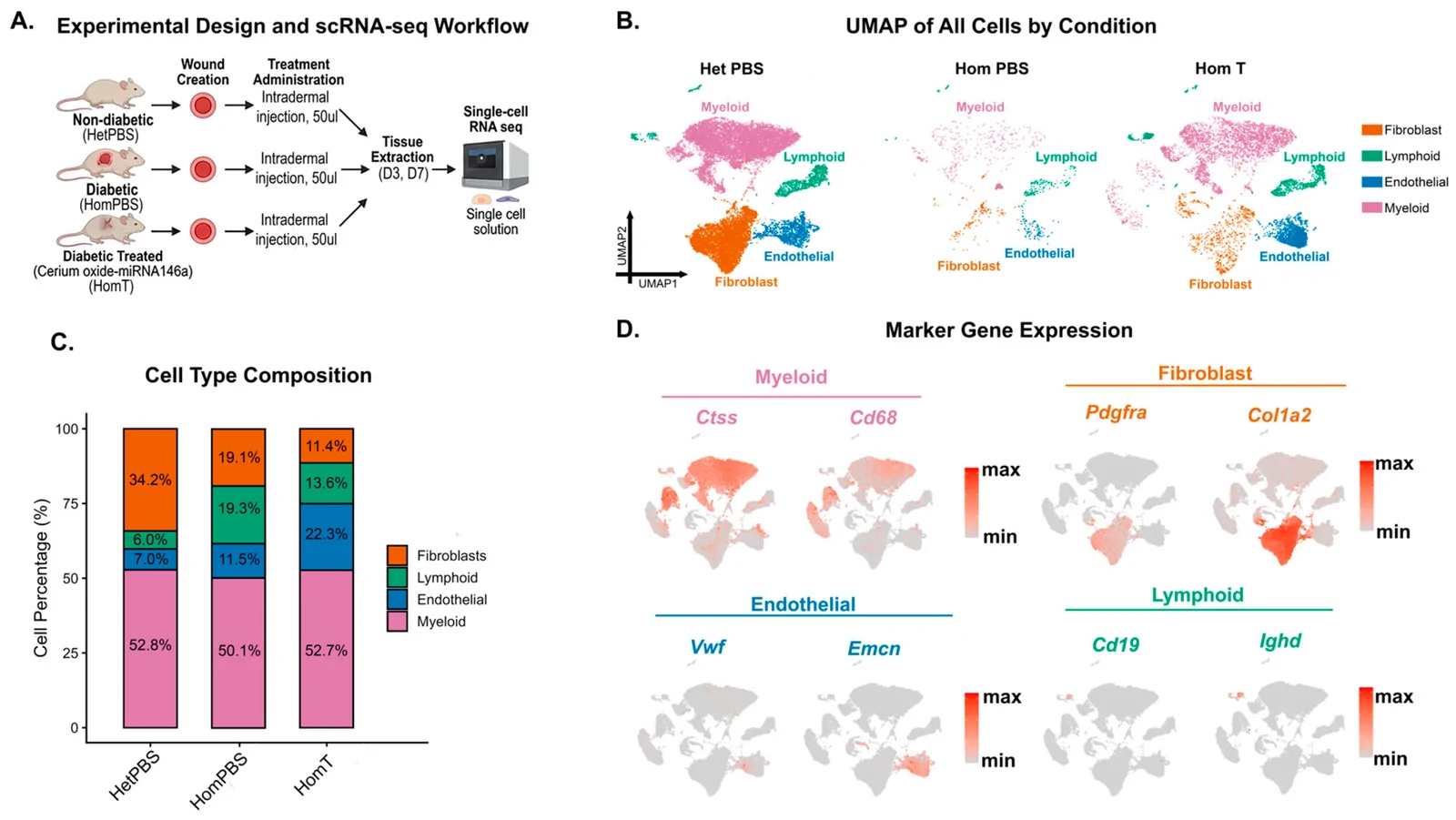
Single-cell transcriptomic profiling of diabetic wounds following CNP-miR146a treatment. (**A**) Experimental design of the diabetic wound-healing model and single-cell RNA-sequencing workflow. Heterozygous non-diabetic control (Het PBS), diabetic PBS-treated control (Hom PBS), and diabetic CNP-miR146a-treated (Hom T) mice underwent full-thickness excisional wounding followed by intradermal administration of PBS or CNP-miR146a. Wound tissues were collected on postoperative Days 3 and 7 for scRNA-seq analysis; the primary analyses shown in Figure 1, Figure 2, Figure 3, Figure 4 and Figure 5 focus on Day 7, while Day 3 is presented as an exploratory temporal comparison in Supplementary Figure S1. (**B**) UMAP visualization of Day 7 wound-derived cells by experimental condition, showing the major annotated myeloid, fibroblast, endothelial, and lymphoid populations. (**C**) Relative representation of major cell populations among captured Day 7 cells across experimental groups. (**D**) Feature plots showing representative canonical marker genes used for cell-type annotation, including *Ctss* and *Cd68* (myeloid), *Pdgfra* and *Col1a2* (fibroblast), *Vwf* and *Emcn* (endothelial), and *Cd19* and *Ighd* (lymphoid).

To characterize cellular responses to CNP-miR146a treatment, we performed single-cell RNA sequencing of wound tissues collected from Het PBS, Hom PBS, and Hom T mice (Figure 1A). Following quality control, integration, and unsupervised clustering, four major cellular populations were identified, including myeloid cells, fibroblasts, endothelial cells, and lymphoid cells (Figure 1B). All major populations were detected across experimental groups.

Visualization of individual conditions demonstrated substantial remodeling of the wound cellular composition across healthy, diabetic, and CNP-miR146a-treated wounds (Figure 1B). Descriptive cell-type composition analysis showed that the overall myeloid compartment remained relatively stable across groups, representing 52.8%, 50.2%, and 52.7% of cells in Het PBS, Hom PBS, and Hom T wounds, respectively (Figure 1C). While this compartment remained relatively stable, diabetic wounds exhibited a pronounced reduction in fibroblasts, which decreased from 34.2% in Het PBS to 19.1% in Hom PBS and remained lower following CNP-miR146a treatment at 11.4%. Lymphoid cells increased in untreated diabetic wounds compared with healthy controls, rising from 6.0% to 19.3%, but decreased after treatment to 13.6%. Endothelial cells represented 7.0%, 11.5%, and 22.3% of captured cells in the pooled Het PBS, Hom PBS, and Hom T datasets, respectively, with the Hom T dataset showing the highest relative representation of endothelial cells. These pooled data indicate that diabetes and CNP-miR146a treatment reshape the wound microenvironment in distinct ways, with treatment associated with a strong enrichment of endothelial cells and partial normalization of lymphoid expansion, while fibroblast representation remains reduced relative to healthy wounds.

Canonical marker analysis confirmed cell-type identities (Figure 1D). Myeloid cells expressed *Ctss* and *Cd68*, fibroblasts expressed *Pdgfra* and *Col1a2*, endothelial cells expressed *Vwf* and *Emcn*, and lymphoid cells expressed *Cd19* and *Ighd*. Together, these findings indicate that diabetes substantially remodels the wound cellular landscape. Because tissues from animals within each experimental group were pooled for scRNA-seq library generation, these cell-type proportions are descriptive and do not represent independent animal-level measurements; therefore, statistical inference regarding treatment-associated changes in cell-type abundance was not performed. Despite the continued lower relative representation of fibroblasts compared with healthy controls, the pooled CNP-miR146a-treated dataset showed a higher relative representation of endothelial cells and alterations in immune-cell composition compared with untreated diabetic wounds. Given the growing evidence implicating ferroptosis in diabetic wound pathology, we next investigated whether these descriptive differences in cellular composition were accompanied by alterations in ferroptosis-associated transcriptional programs across the wound microenvironment.

To contextualize for the Day 7 analyses, we additionally examined the Day 3 wound datasets (Supplementary Figure S1). At Day 3, myeloid cells constituted the predominant cellular population across all experimental groups, representing 80.0%, 74.5%, and 84.4% of cells in Het PBS, Hom PBS, and Hom T wounds, respectively (Supplementary Figure S1A,B). Day 3 analysis also revealed differences in ferroptosis-associated transcriptional signatures among experimental conditions, including pro-ferroptotic, anti-ferroptotic, and iron-homeostasis module scores (Supplementary Figure S1C). These early-stage observations provide temporal context for the primary mechanistic analyses performed on Day 7.

### 3.2. CNP-miR146a Is Associated with Ferroptosis-Protective and Iron-Homeostasis Transcriptional Programs

**Figure 2:**
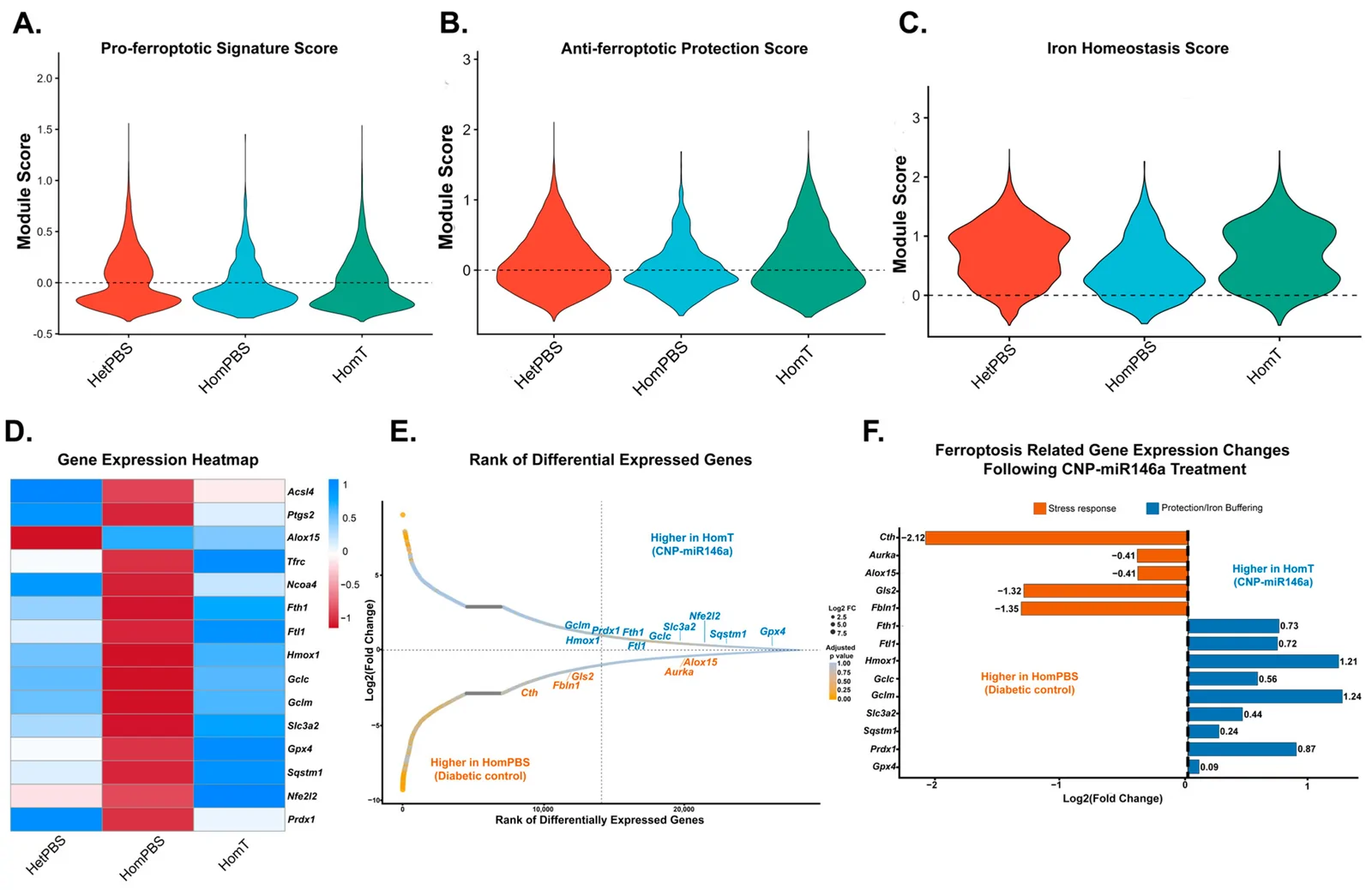
CNP-miR146a is associated with ferroptosis-related antioxidant and iron homeostasis transcriptional programs in diabetic wounds. (**A**–**C**) Violin plots showing the distributions of pro-ferroptotic, anti-ferroptotic protection, and iron homeostasis module scores across all wound cells from pooled Het PBS, Hom PBS, and Hom T datasets. (**D**) Heatmap showing scaled average expression of representative genes associated with ferroptosis regulation, antioxidant defense, and iron homeostasis across experimental groups. (**E**) Ranking of ferroptosis-associated genes according to treatment-associated expression differences between CNP-miR146a-treated diabetic wounds (Hom T) and untreated diabetic wounds (Hom PBS). Genes preferentially expressed in Hom T and Hom PBS are shown in blue and orange, respectively. (**F**) Expression differences of selected ferroptosis-associated genes between Hom T and Hom PBS wounds.

To characterize the impact of CNP-miR146a treatment on ferroptosis-associated pathways within the wound microenvironment, we evaluated global ferroptosis-related transcriptional programs across all cells from Het PBS, Hom PBS, and Hom T wounds (Figure 2). Module score analysis revealed differences in ferroptosis-associated transcriptional signatures among experimental groups. Pro-ferroptotic signature scores differed among three groups, indicating broader alterations in ferroptosis-associated transcriptional programs within the diabetic wound microenvironment (Figure 2A). In parallel, untreated diabetic wounds exhibited lower anti-ferroptotic protection and iron homeostasis scores compared with healthy controls (Figure 2B,C). In contrast, CNP-miR146a treatment increased both anti-ferroptotic protection and iron homeostasis scores relative to untreated diabetic wounds, shifting these transcriptional signatures toward those observed in non-diabetic wounds.

To further characterize these transcriptional changes, we examined the expression of representative genes associated with lipid peroxidation, antioxidant defense, and iron homeostasis across experimental groups (Figure 2D). Hom PBS exhibited reduced expression of multiple genes associated with ferroptosis protection and cellular antioxidant defense, including *Fth1*, *Ftl1*, *Hmox1*, *Gclc*, *Gclm*, *Slc3a2*, *Gpx4*, *Sqstm1*, *Nfe2l2*, and *Prdx1*, compared with healthy controls. In contrast, Hom T demonstrated increased expression of genes involved in iron sequestration, glutathione metabolism, and NRF2-mediated antioxidant responses. In addition to reduced expression of antioxidant and iron-buffering genes, untreated diabetic wounds exhibited reduced expression of several ferroptosis-associated genes, including *Acsl4*, *Ptgs2*, *Tfrc*, and *Ncoa4*. Expression of these genes was increased following CNP-miR146a treatment, although the magnitude of the change varied among individual genes. These findings suggest broad dysregulation of ferroptosis-associated transcriptional programs in diabetic wounds rather than uniform activation of all ferroptosis-related genes.

Differential expression ranking analysis further demonstrated a treatment-associated shift toward ferroptosis-related transcriptional programs in CNP-miR146a-treated wounds (Figure 2E). Genes associated with antioxidant defense and iron homeostasis were preferentially enriched in treated wounds, whereas untreated diabetic wounds exhibited a transcriptional profile consistent with impaired ferroptosis resistance and disrupted iron homeostasis. Consistent with these findings, direct comparison of selected ferroptosis-related genes revealed increased expression of *Hmox1*, *Gclc*, *Gclm*, *Fth1*, *Ftl1*, *Slc3a2*, *Sqstm1*, and *Prdx1* following treatment, while expression of several pro-ferroptotic mediators was reduced (Figure 2F).

Together, these data demonstrate lower anti-ferroptotic protection and iron homeostasis signatures in diabetic wounds. CNP-miR146a treatment increased ferroptosis-protective module scores and showed higher expression of genes associated with antioxidant defense, glutathione metabolism, and iron buffering. These transcriptional changes were observed across the wound microenvironment and were accompanied by enrichment of NRF2-associated cytoprotective programs.

### 3.3. CNP-miR146a Is Associated with NRF2-Related Ferroptosis-Protective Programs in Myeloid Cells

**Figure 3:**
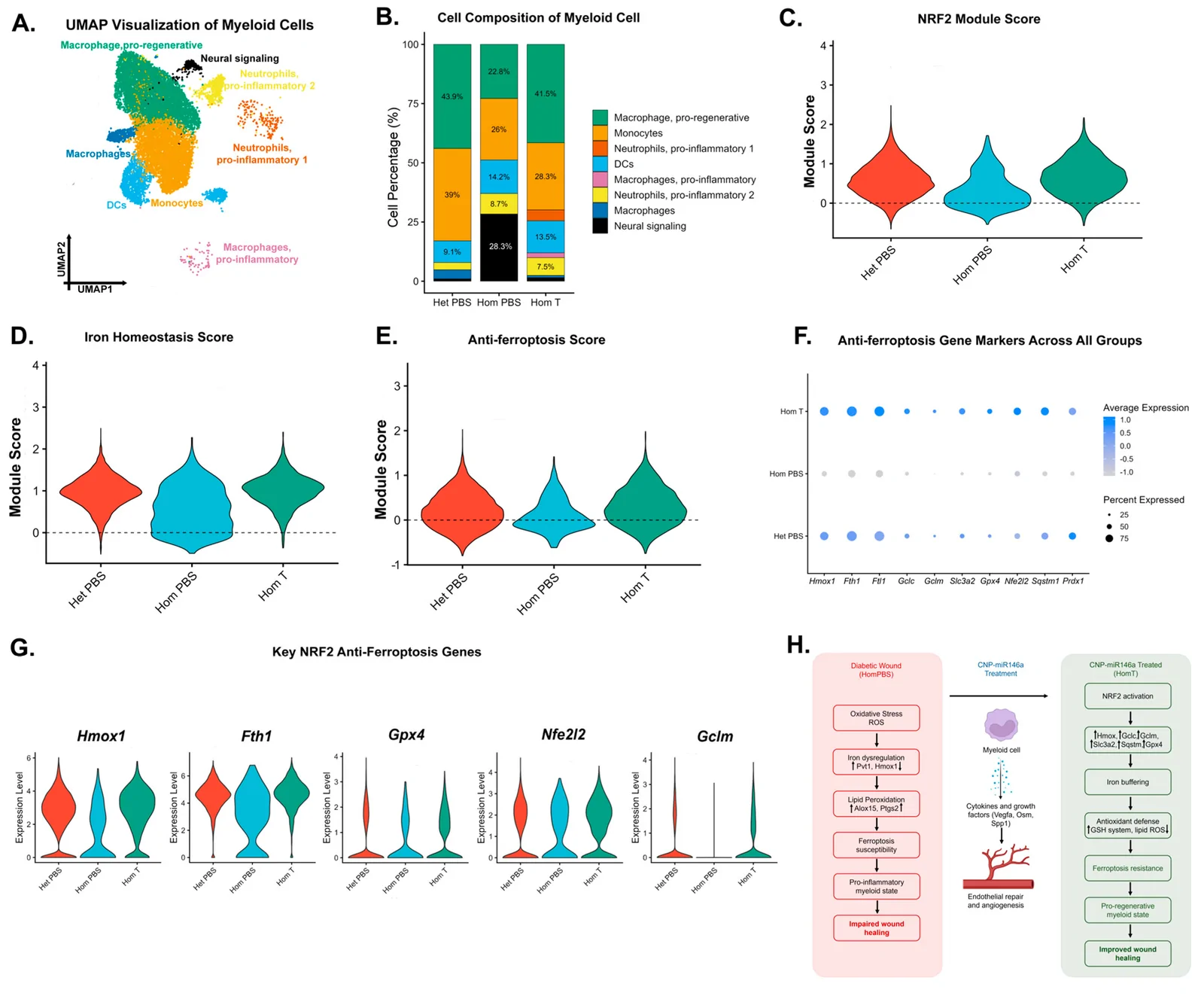
CNP-miR146a is associated with NRF2-related ferroptosis-protective transcriptional programs and reparative myeloid states in diabetic wounds. (**A**) UMAP visualization of myeloid cell subpopulations across experimental groups. (**B**) Relative representation of myeloid subpopulations among cells captured in the pooled Het PBS, Hom PBS, and Hom T datasets. (**C**–**E**) Violin plots showing the distributions of myeloid-specific NRF2-associated antioxidant, iron-homeostasis, and anti-ferroptotic protection module scores across experimental groups. (**F**) Dot plot showing expression of representative NRF2-associated and ferroptosis-related genes across myeloid cells. (**G**) Violin plots showing expression distributions of selected antioxidant and ferroptosis-protective genes (*Hmox1*, *Fth1*, *Gpx4*, *Nfe2l2*, and *Gclm*) across experimental groups. (**H**) Schematic summarizing the proposed association between CNP-miR146a treatment, NRF2-associated antioxidant and iron-homeostasis transcriptional programs, ferroptosis-protective gene expression, and reparative myeloid states.

To determine how CNP-miR146a treatment is associated with myeloid cell states during diabetic wound healing, we isolated the myeloid compartment and performed subclustering analysis to identify transcriptionally distinct myeloid populations (Figure 3A). Unsupervised clustering revealed multiple myeloid states, including monocytes, dendritic cells, neutrophils, pro-inflammatory macrophages, and pro-regenerative macrophages. Descriptive comparison of cellular composition across experimental groups revealed differences in the distribution of myeloid populations (Figure 3B). Hom PBS exhibited a marked reduction in pro-regenerative macrophages, which decreased from 43.9% in healthy wounds to 22.8%, accompanied by expansion of a neural signaling-associated myeloid population and increased dendritic cell representation. In contrast, the Hom T dataset showed higher relative representation of pro-regenerative macrophages at 41.5% and a markedly reduced neural signaling-associated population while maintaining similar levels of monocytes and dendritic cells. These findings indicate that the pooled CNP-miR146a-treated dataset exhibited a myeloid composition more closely resembling the healthy control dataset, particularly with respect to the representation of pro-regenerative macrophages.

Because oxidative stress and ferroptosis have emerged as key drivers of diabetic wound pathology, we next examined NRF2-associated antioxidant programs within myeloid cells. NRF2 module scores were lower in untreated diabetic wounds compared with non-diabetic controls (Figure 3C). Importantly, CNP-miR146a treatment was associated with higher NRF2 module scores relative to untreated diabetic controls, shifting the transcriptional signature toward levels observed in healthy controls. Consistent with this finding, iron homeostasis scores were lower in untreated diabetic wounds, with a substantial fraction of cells exhibiting low scores (Figure 3D). Following CNP-miR146a treatment, the distribution shifted toward higher scores and became more similar to that observed in healthy wounds. These findings indicate treatment-associated increases in NRF2-related antioxidant and iron-homeostasis transcriptional programs within the myeloid compartment.

To further assess ferroptosis-associated transcriptional states, we calculated anti-ferroptosis module scores using genes involved in glutathione metabolism, antioxidant defense, and lipid peroxide detoxification. Untreated diabetic wounds displayed lower anti-ferroptosis module scores, whereas treatment exhibited higher anti-ferroptosis scores that were comparable with or exceeded those observed in healthy controls (Figure 3E). These findings indicate that treatment is associated with a shift toward a more ferroptosis-protective transcriptional state in wound-associated myeloid cells.

Analysis of individual ferroptosis-regulating genes further supported treatment-associated changes in NRF2-related cytoprotective transcriptional programs (Figure 3F–G, Supplementary Figure S3). Key antioxidant and iron-sequestration genes, including *Hmox1*, *Fth1*, *Ftl1*, *Gclc*, *Gclm*, *Slc3a2*, *Gpx4*, *Nfe2l2*, *Sqstm1*, and *Prdx1*, exhibited increased expression following treatment compared with untreated diabetic controls. Several of these genes were reduced in untreated diabetic wounds and showed increased expression following CNP-miR146a treatment. In particular, increased expression of *Hmox1*, *Fth1*, and *Ftl1* was consistent with enhanced transcriptional programs associated with iron handling, while elevated *Gclc*, *Gclm*, *Slc3a2*, and *Gpx4* expression was consistent with enhanced glutathione synthesis and protection against lipid peroxidation. Together, these transcriptional changes support an NRF2-associated ferroptosis-protective gene-expression program in CNP-miR146a-treated myeloid cells. Collectively, these findings demonstrate increased NRF2-related, iron-homeostasis, and anti-ferroptosis transcriptional signatures within myeloid cells following CNP-miR146a treatment (Figure 3H).

### 3.4. CNP-miR146a Is Associated with Enhanced Endothelial Regenerative and Angiogenic Transcriptional Programs

**Figure 4:**
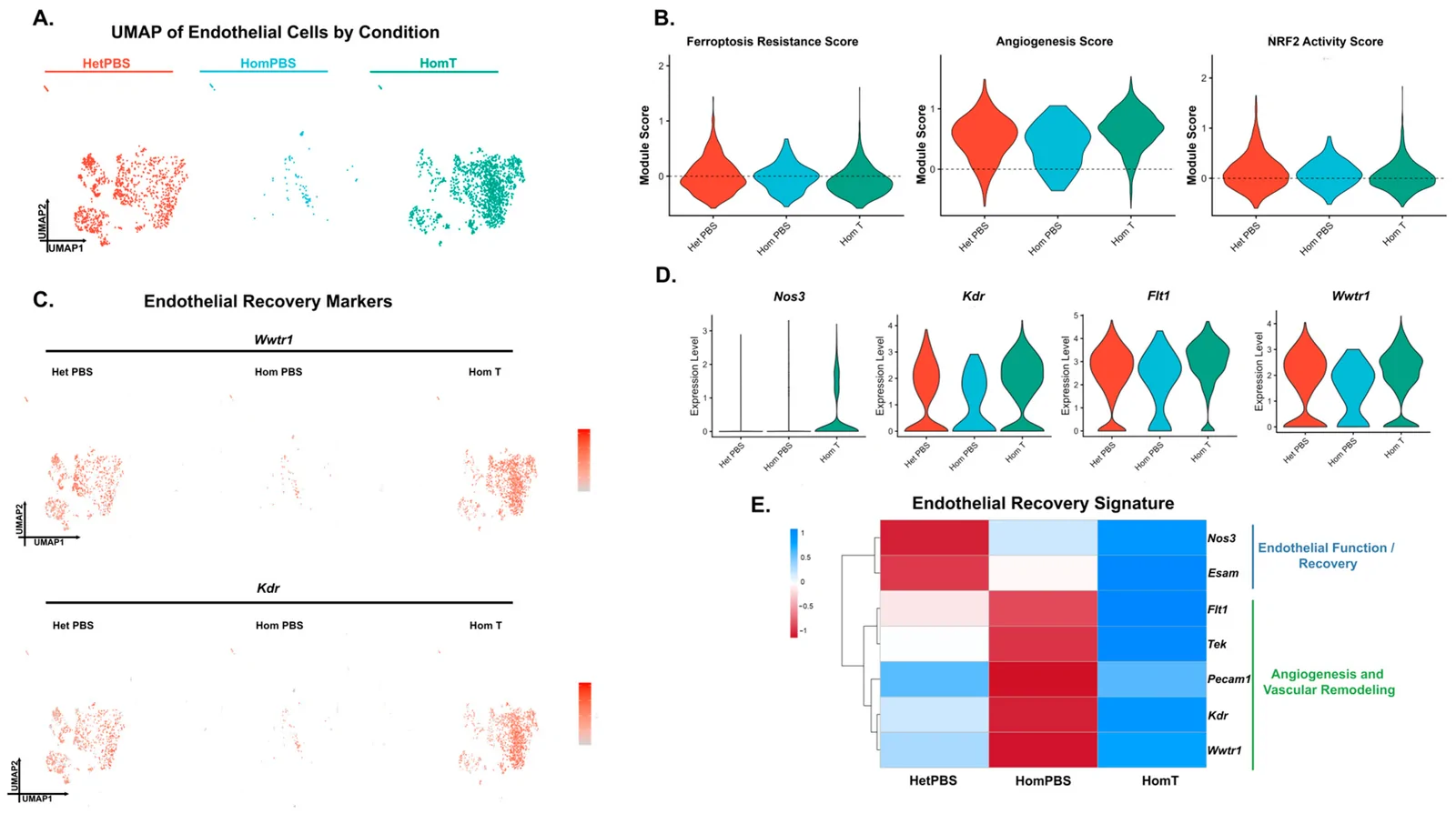
CNP-miR146a is associated with enhanced endothelial regenerative and angiogenic transcriptional programs in diabetic wounds. (**A**) UMAP visualization of endothelial cells from pooled Het PBS, Hom PBS, and Hom T datasets. (**B**) Violin plots showing the distributions of endothelial ferroptosis-protective, angiogenesis-associated, and NRF2-associated transcriptional module scores across experimental groups. (**C**) Feature plots showing expression of representative endothelial repair-associated genes, including *Wwtr1* and *Kdr.* (**D**) Violin plots showing expression distributions of endothelial repair- and angiogenesis-associated genes (*Nos3*, *Kdr*, *Flt1*, and *Wwtr1*) across experimental groups. (**E**) Heatmap showing expression patterns of representative genes associated with endothelial repair, maintenance, and angiogenesis across experimental groups.

Given the critical role of endothelial dysfunction in diabetic wound pathology, we next investigated endothelial cell transcriptional programs associated with angiogenesis, oxidative stress, and vascular repair following CNP-miR146a treatment. Endothelial cells were isolated from the integrated single-cell dataset and analyzed separately across experimental groups (Figure 4A). UMAP visualization demonstrated endothelial populations in all conditions, with a higher representation of endothelial cells in the pooled CNP-miR146a-treated wounds relative to untreated diabetic controls.

To characterize endothelial stress- and repair-associated transcriptional programs, ferroptosis-protective, angiogenesis, and NRF2-associated module scores were evaluated across experimental groups (Figure 4B). Endothelial cells from untreated diabetic wounds exhibited lower angiogenesis and NRF2-associated module scores compared with non-diabetic controls. CNP-miR146a treatment increased angiogenesis module scores relative to untreated diabetic wounds, indicating enrichment of angiogenesis-associated transcriptional programs. Treatment also increased NRF2-related module scores. Although ferroptosis-protective scores exhibited more modest differences, they were consistent with treatment-associated changes in cytoprotective transcriptional programs.

To further characterize endothelial transcriptional responses, we examined the expression of key vascular repair-associated genes. Feature plot analysis demonstrated increased expression of the endothelial recovery markers *Wwtr1* [36] and *Kdr* [22] in treated wounds compared with untreated diabetic controls (Figure 4C). Consistent with these observations, violin plot analysis revealed increased expression of *Nos3*, *Kdr*, *Flt1*, and *Wwtr1* in endothelial cells following CNP-miR146a treatment (Figure 4D). *Nos3* [37], a key regulator of nitric oxide production and endothelial function, was markedly elevated following treatment. Similarly, increased expression of *Kdr* and *Flt1* [38], which encode major VEGF receptors, was consistent with enrichment of angiogenesis-associated transcriptional programs. Increased *Wwtr1* expression further supported treatment-associated changes in transcriptional programs related to endothelial regeneration and vascular remodeling.

To evaluate broader endothelial transcriptional responses, we generated an endothelial recovery signature composed of genes associated with vascular function, endothelial maintenance, and angiogenesis (Figure 4E). This analysis revealed a distinct expression pattern between untreated diabetic and CNP-miR146a-treated endothelial cells. Genes associated with endothelial function and recovery, including *Nos3*, *Esam*, and *Flt1*, showed increased expression following treatment, together with angiogenesis-associated genes including *Tek*, *Pecam1*, *Kdr*, and *Wwtr1*. Together, these findings demonstrate that CNP-miR146a treatment is associated with coordinated changes in endothelial angiogenesis-, NRF2-, and vascular repair-associated transcriptional programs, supporting an endothelial transcriptional state consistent with vascular repair.

Collectively, these data demonstrate prominent treatment-associated transcriptional changes within endothelial cells. Following CNP-miR146a treatment, endothelial cells exhibited higher NRF2-associated and ferroptosis-protective transcriptional scores together with enrichment of angiogenesis- and vascular repair-associated gene-expression programs.

### 3.5. CNP-miR146a Is Associated with a Repair-Related Myeloid Trajectory and Increased Predicted Immune–Vascular Communication

**Figure 5:**
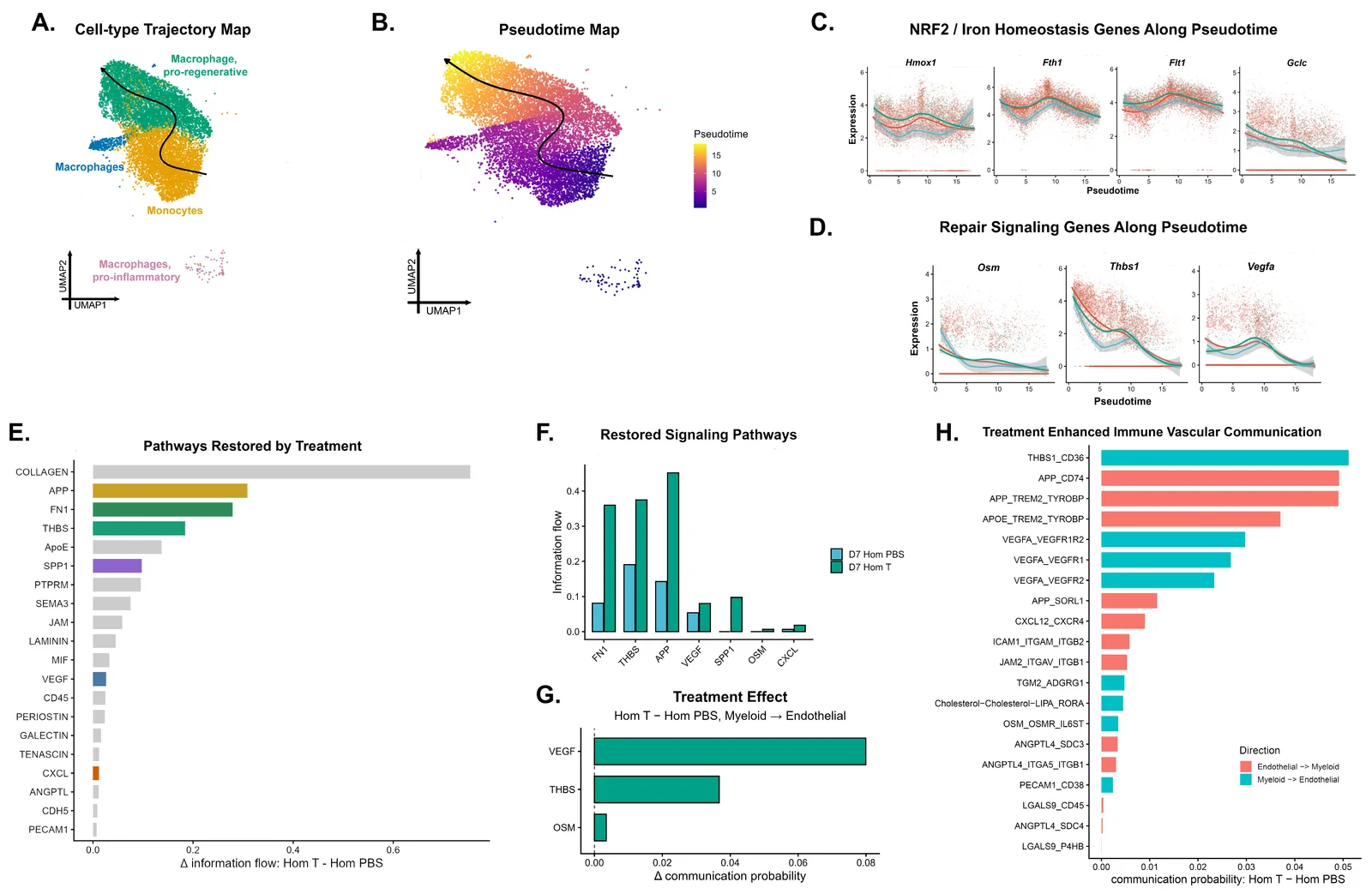
CNP-miR146a is associated with a repair-associated myeloid trajectory and increased predicted immune–vascular communication in diabetic wounds. (**A**) Slingshot-inferred trajectories of wound myeloid cells, including the repair-associated lineage from monocytes toward pro-regenerative macrophages. (**B**) Pseudotime distribution of myeloid cells along the repair-associated trajectory. (**C**) Smoothed expression trends of representative NRF2-associated antioxidant and iron homeostasis genes (*Hmox1*, *Fth1*, *Ftl1*, and *Gclc*) along pseudotime. (**D**) Smoothed expression trends of representative repair-associated genes (*Osm*, *Thbs1*, and *Vegfa*) along pseudotime. (**E**) CellChat-inferred signaling pathways exhibiting increased information flow in CNP-miR146a-treated wounds relative to untreated diabetic wounds. (**F**) Predicted pathway-level communication activity in untreated diabetic and CNP-miR146a-treated wounds. (**G**) Treatment-associated differences in predicted myeloid-to-endothelial communication pathways, including VEGF and THBS signaling. (**H**) CellChat-inferred ligand–receptor communication probabilities between myeloid and endothelial populations. CellChat-derived communication networks are based on ligand and receptor transcript expression and represent predicted interactions rather than experimentally validated signaling events.

To investigate treatment-associated changes in myeloid cell state transitions during diabetic wound repair, we performed pseudotime trajectory analysis of the Day 7 myeloid compartment. Slingshot inferred three trajectories originating from monocytes and terminating in pro-regenerative macrophages, macrophages, or pro-inflammatory macrophages. Subsequent repair-associated pseudotime analyses focused on the lineage connecting monocytes to pro-regenerative macrophages (Figure 5A). While pro-inflammatory macrophages occupied a transcriptionally distinct branch, the repair-associated trajectory represented a continuum from monocytes toward pro-regenerative macrophages, suggesting a transcriptional maturation program associated with regenerative myeloid states within the wound microenvironment. Pseudotime mapping demonstrated progressive transcriptional changes along this lineage, with cells distributed across early, intermediate, and late transcriptional states (Figure 5B).

To determine how ferroptosis- and oxidative-stress-associated transcriptional programs varied along the repair-associated trajectory, we examined the expression dynamics of NRF2-associated antioxidant, iron-homeostasis, and vascular-repair genes across pseudotime (Figure 5C). Several treatment-responsive genes exhibited distinct expression patterns throughout the trajectory. *Hmox1* and *Fth1*, key mediators of oxidative stress adaptation and iron sequestration, showed consistently higher expression across portions of the pseudotime continuum in CNP-miR146a-treated wounds compared with untreated diabetic controls. Similarly, *Gclc*, a critical regulator of glutathione synthesis and antioxidant defense, showed elevated expression during early and intermediate pseudotime states. In addition, expression of *Flt1*, a VEGF receptor associated with angiogenic signaling, was increased along portions of the trajectory following treatment. Collectively, these transcriptional patterns indicate that CNP-miR146a treatment is associated with altered antioxidant, iron homeostasis, and vascular-repair gene expression programs along the repair-associated myeloid trajectory.

Next, we examined the expression dynamics of repair-associated signaling molecules along pseudotime (Figure 5D). Genes involved in tissue regeneration and vascular remodeling, including *Osm*, *Thbs1*, and *Vegfa*, exhibited distinct temporal expression patterns along the trajectory. *Osm* and *Thbs1* were most highly expressed during early pseudotime states and gradually declined as cells progressed along the trajectory, whereas *Vegfa* expression peaked at intermediate pseudotime. Treatment-associated differences were also observed for several of these genes across specific pseudotime states, including *Thbs1* and *Vegfa*. These findings suggest that progression along the repair-associated myeloid trajectory is accompanied by dynamic expression of genes associated with antioxidant defense, iron handling, and vascular repair.

Given the treatment-associated endothelial transcriptional changes observed in Figure 4, we next used CellChat to infer potential intercellular communication networks between cellular compartments based on ligand and receptor transcript expression. Comparison of inferred communication networks between untreated diabetic and CNP-miR146a-treated wounds revealed treatment-associated differences in predicted pathway-level information flow (Figure 5E). Several pathways, including COLLAGEN, APP, FN1, THBS, SPP1, LAMININ, and VEGF, exhibited increased inferred information flow in treated wounds, suggesting altered communication programs associated with extracellular matrix remodeling and vascular repair. Quantitative analysis of CellChat-inferred pathway activity showed increased predicted signaling activity for several repair-associated pathways in treated wounds compared with diabetic controls (Figure 5F). Notably, FN1, THBS, and APP exhibited inferred communication activity in treated wounds compared with diabetic controls. Differential communication analysis also predicted treatment-associated increases in VEGF, THBS, and OSM signaling networks (Figure 5G), with VEGF exhibiting a prominent increase in inferred information flow. These transcriptome-based predictions were consistent with the endothelial repair-associated transcriptional patterns observed in Figure 4 but do not establish functional pathway activation.

To identify candidate interactions contributing to these predicted communication networks, we evaluated CellChat-inferred ligand–receptor communication probabilities between myeloid and endothelial populations (Figure 5H). CNP-miR146a-treated wounds showed increased predicted communication probabilities for several repair-associated ligand–receptor pairs, including VEGFA-VEGFR1, VEGFA-VEGFR2, THBS1-CD36, and OSM-OSMR/IL6ST. Additional treatment-associated differences were predicted for APP-associated, CXCL12-CXCR4, and integrin-mediated communication. Collectively, the trajectory and CellChat analyses indicate that CNP-miR146a treatment is associated with a repair-associated myeloid transcriptional trajectory and with increased predicted immune–vascular communication programs. These CellChat-derived interactions represent transcriptomically inferred candidate communication mechanisms and should not be interpreted as direct experimental evidence of ligand–receptor signaling.

## 4. Discussion

In the present study, we used single-cell transcriptomics, trajectory analysis, and cell–cell communication modeling to characterize the cellular and transcriptional programs associated with CNP-miR146a-mediated diabetic wound repair. CNP-miR146a treatment was associated with enhanced NRF2-related antioxidant, ferroptosis-protective, and iron-homeostasis transcriptional programs in myeloid cells, together with pro-regenerative myeloid states, endothelial angiogenesis-associated programs, and predicted immune–vascular communication. These findings provide new insight into the cellular programs underlying the therapeutic activity of CNP-miR146a and identify ferroptosis-associated and immune–vascular transcriptional networks as candidate mechanisms for further functional investigation.

A major finding of this study is the identification of widespread suppression of ferroptosis-protective pathways within diabetic wounds. Untreated diabetic wounds exhibited reduced anti-ferroptotic protection and iron-homeostasis module scores together with decreased expression of NRF2-associated antioxidant and iron-buffering genes, including *Hmox1*, *Fth1*, *Ftl1*, *Gclc*, *Gclm*, *Slc3a2*, and *Gpx4*. These findings are consistent with accumulating evidence implicating ferroptosis in the pathogenesis of diabetic wound healing defects [39]. Chronic hyperglycemia promotes oxidative stress, lipid peroxidation, mitochondrial dysfunction, and disturbances in iron metabolism that collectively create a microenvironment highly susceptible to ferroptotic injury [40]. More broadly, ferroptosis has also been implicated in other diabetes-associated tissue complications, and pharmacological inhibition of ferroptosis has been shown to attenuate diabetic tissue injury, further supporting a link between metabolic oxidative stress and ferroptosis-associated pathology [41]. Our findings extend these observations by identifying ferroptosis-associated transcriptional alterations across multiple cellular populations within the diabetic wound microenvironment that were broadly altered following CNP-miR146a treatment. Interestingly, not all ferroptosis-associated genes followed the same expression pattern. Several genes commonly associated with ferroptotic processes, including *Acsl4*, *Ptgs2*, *Tfrc*, and *Ncoa4*, were reduced in untreated diabetic wounds relative to healthy controls and exhibited increased expression following CNP-miR146a treatment. While these genes are frequently described as mediators or markers of ferroptosis, they also participate in lipid metabolism, iron uptake, and ferritin turnover. Their reduced expression may therefore reflect broader impairment of lipid metabolic, iron-handling, and cellular stress-response pathways within the chronic diabetic wound microenvironment rather than coordinated upregulation of all canonical ferroptosis-associated genes. This observation highlights the complexity of ferroptosis regulation in chronic diabetic wounds and suggests that ferroptosis-associated dysfunction may involve disruption of multiple interconnected metabolic and iron-homeostasis programs [42,43,44,45]. Importantly, the present study does not directly demonstrate ferroptosis or ferroptosis inhibition. The ferroptosis-related findings are based on transcriptional signatures associated with antioxidant defense, iron homeostasis, and ferroptosis susceptibility. Direct biochemical and functional measurements, including lipid peroxidation, intracellular labile iron, GSH/GSSG ratio, GPX4 activity, ROS, and MDA/4-HNE, were not performed and will be required to determine whether these transcriptional changes correspond to functional alterations in ferroptosis in wound myeloid cells.

Myeloid cells exhibited prominent treatment-associated transcriptional changes and were therefore examined in greater detail. Untreated diabetic wounds demonstrated a marked reduction in pro-regenerative macrophages, decreasing from 43.9% in healthy wounds to 22.8%, accompanied by reduced NRF2-associated transcriptional activity, diminished anti-ferroptotic signaling, and impaired iron-homeostasis programs. In contrast, CNP-miR146a showed a higher representation of pro-regenerative macrophages at 41.5% while simultaneously showing increased NRF2, anti-ferroptosis, and iron-homeostasis module scores. NRF2 is increasingly recognized as a master regulator of ferroptosis resistance through its ability to coordinate glutathione metabolism, antioxidant defense, and intracellular iron homeostasis [46]. These treatment-associated transcriptional changes suggest that CNP-miR146a is associated with a ferroptosis-protective myeloid transcriptional phenotype within the oxidative diabetic wound environment [47]. These observations are consistent with previous reports demonstrating anti-ferroptotic and cytoprotective effects of both NRF2 activation and cerium oxide nanoparticles [48,49]. However, NRF2 activity was inferred from transcriptional signatures rather than directly measured or experimentally perturbed. Thus, the present findings demonstrate an association between CNP-miR146a treatment and NRF2-related transcriptional programs but do not establish a causal requirement for NRF2 signaling.

Trajectory analysis further revealed that treatment-associated ferroptosis-protective transcriptional programs occurred along a repair-associated myeloid trajectory. Across the inferred pseudotime trajectory, antioxidant and iron-buffering genes exhibited distinct expression patterns, while regenerative signaling molecules, including *Vegfa*, *Osm*, and *Thbs1*, showed dynamic expression changes. Notably, *Hmox1*, *Fth1*, and *Gclc* remained elevated across large portions of the trajectory, consistent with sustained expression of antioxidant- and iron-homeostasis-associated genes across reparative macrophage-associated transcriptional states. These findings suggest that ferroptosis-protective transcriptional programs are accompanied by expression of regenerative signaling molecules, including *Vegfa*, *Osm*, and *Thbs1*, which may contribute to tissue repair [50]. Together, these data suggest that ferroptosis-associated transcriptional programs may be linked to cellular programs involved in wound regeneration.

Importantly, pseudotime analysis infers relationships among transcriptional states and does not establish actual temporal differentiation or lineage conversion. Therefore, the trajectories identified here should be interpreted as candidate transcriptional state transitions rather than demonstrated monocyte-to-macrophage differentiation pathways. Temporal validation and lineage-tracing studies will be required to determine whether these inferred trajectories correspond to actual myeloid-cell differentiation in vivo.

A second major finding of this study is the identification of prominent treatment-associated transcriptional changes within endothelial cells. Endothelial dysfunction is a hallmark of diabetic wounds and contributes directly to impaired angiogenesis, reduced tissue perfusion, and delayed tissue repair [51]. Following CNP-miR146a treatment, endothelial cells exhibited higher angiogenesis-associated and NRF2-associated transcriptional scores, together with increased expression of genes associated with endothelial function and vascular signaling, including *Nos3*, *Kdr* (VEGFR2), *Flt1* (VEGFR1), and *Wwtr1*. In addition, the proportion of endothelial cells in the dataset increased from 11.5% of cells in untreated diabetic wounds to 22.3% following treatment. However, because tissues from animals within each experimental group were pooled for scRNA-seq library generation, these cell-type proportions represent descriptive differences within the pooled datasets and cannot establish animal-level differences in cellular abundance. Independent biological replicates will be required to statistically validate treatment-associated changes in endothelial and other cell-type proportions. These findings should be interpreted as angiogenesis-associated transcriptional responses rather than direct functional evidence of angiogenesis, as endothelial proliferation and migration were not assessed in the present study. However, our previous studies using the CNP-miR146a diabetic wound model demonstrated increased VEGF expression and CD31-positive staining following treatment [25], providing complementary tissue-level evidence of a vascular response. These observations are consistent with growing evidence that endothelial cells are vulnerable to oxidative stress and ferroptosis-associated injury under diabetic conditions [52]. The observed NRF2-associated endothelial transcriptional response may therefore be relevant to vascular repair; however, the present data do not establish whether this response reflects a direct effect of CNP-miR146a on endothelial cells or an indirect effect mediated through other wound-cell populations. We therefore next examined whether these treatment-associated transcriptional changes were accompanied by alterations in predicted intercellular communication networks.

Another important finding of this study is the association between treatment-related transcriptional changes and predicted immune–vascular communication. CellChat analysis inferred treatment-associated increases in communication probabilities across several repair-related pathways, including VEGF, THBS, FN1, APP, COLLAGEN, and LAMININ. At the ligand–receptor level, CNP-miR146a was associated with increased predicted communication probabilities for several pro-regenerative interactions, including VEGFA-VEGFR1, VEGFA-VEGFR2, THBS1-CD36, and OSM-OSMR/IL6ST. These predicted interactions are consistent with the endothelial angiogenesis-associated transcriptional programs observed in treated wounds [21,23,53]. Together, these findings suggest that treatment-associated changes in antioxidant and iron homeostasis transcriptional programs within myeloid cells occur alongside predicted changes in regenerative immune–vascular communication. Macrophage-endothelial crosstalk is increasingly recognized as a critical regulator of angiogenesis, vascular remodeling, and tissue repair, and disruption of these interactions contributes to impaired healing in chronic wounds [54]. While previous studies have largely focused on direct effects of oxidative stress on individual cell populations, our results indicate that ferroptosis may influence tissue repair at the multicellular level through regulation of intercellular signaling networks [40]. This observation provides a potential explanation for why therapies targeting oxidative stress alone often demonstrate limited regenerative efficacy. Importantly, CellChat-derived ligand–receptor networks are computationally inferred from transcriptomic expression and therefore represent candidate communication mechanisms rather than experimentally established signaling interactions. Although our previous studies using the same CNP-miR146a diabetic wound model demonstrated increased VEGF expression, CD31-positive staining, collagen deposition, and improved wound architecture following treatment [25], these tissue-level findings provide biological context for, but do not directly validate, the specific ligand–receptor interactions predicted here. Future protein-level and functional studies will be required to validate selected signaling pathways.

The findings of the present study are built directly upon our previous work [25] demonstrating that CNP-miR146a accelerates diabetic wound healing, improves histological wound architecture, enhances collagen deposition, and increases expression of pro-repair genes, including *Vegfa*, *Tgfb1*, *Col1a2*, and *Col3a1*. While those studies established the therapeutic efficacy of CNP-miR146a at the tissue level, the cellular mechanisms responsible for these regenerative effects remained unclear. The present single-cell analysis extends these findings by resolving treatment-associated NRF2-related ferroptosis-protective programs within the myeloid compartment and linking these changes to endothelial regenerative programs and predicted immune–vascular communication. Rather than identifying NRF2–ferroptosis regulation itself as a novel mechanism, our findings provide cell-resolved transcriptional context for how this previously implicated pathway may participate in the response to CNP-miR146a treatment. Importantly, the increased VEGF expression, enhanced collagen deposition, and improved wound architecture previously observed following CNP-miR146a treatment are consistent with the endothelial regenerative and immune–vascular signaling programs identified in the present study. Because the primary mechanistic analyses focused on postoperative Day 7, with Day 3 included as an exploratory temporal comparison, future longitudinal studies incorporating additional time points and functional validation will be needed to define the temporal relationship between treatment-associated myeloid transcriptional changes, endothelial responses, and tissue regeneration.

The multifaceted nature of these regenerative responses likely reflects the complementary mechanisms of the CNP-miR146a platform. Cerium oxide nanoparticles possess catalytic antioxidant activity that enables repeated scavenging of reactive oxygen species and modulation of cellular redox signaling, while miR-146a suppresses inflammatory activation through inhibition of IRAK1- and TRAF6-dependent NF-κB signaling [25,55]. The potential relevance of antioxidant modulation to wound repair is also supported by studies evaluating other antioxidant-based therapeutic strategies. For example, Husni et al. demonstrated antioxidant activity of *Citrus medica* and *Citrus × microcarpa* essential oils together with effects on fibroblast proliferation, migration, and in vitro wound closure [56]. Although mechanistically distinct from CNP-miR146a, these findings support the broader concept that modulation of oxidative stress may contribute to a cellular environment favorable for wound repair. Together, these mechanisms may simultaneously reduce oxidative stress, limit ferroptosis susceptibility, attenuate chronic inflammation, and restore regenerative communication networks within the diabetic wound microenvironment [25,50].

An important limitation of the present study is that the scRNA-seq experiment included the CNP-miR146a formulation and PBS controls but did not include CNP-only or free miR-146a treatment groups. Therefore, the current transcriptomic data cannot distinguish CNP-mediated redox regulation from miR-146a-mediated biological effects or determine whether the observed NRF2-associated, ferroptosis-protective, and inflammatory transcriptional responses reflect the individual or combined actions of these components. Although the individual components have been evaluated in our previous diabetic wound studies [25], these prior findings do not substitute for component-specific controls within the present scRNA-seq experiment. Future transcriptomic studies incorporating CNP-only and free miR-146a groups will be required to resolve the individual and combined contributions of these components to the observed transcriptional responses.

An additional limitation is that cell-specific uptake and intracellular delivery of miR-146a were not directly quantified in the present study. Therefore, the transcriptional responses observed in myeloid and endothelial populations cannot be interpreted as evidence of direct CNP-miR146a internalization by these cells. The observed responses may reflect direct treatment effects, indirect effects mediated through intercellular signaling, or a combination of both. Future studies incorporating cell-specific localization and quantification of intracellular miR-146a will be required to determine CNP-miR146a delivery to macrophages/monocytes and endothelial cells.

An additional limitation is that tissues from animals within each experimental group were pooled prior to scRNA-seq analysis; consequently, the resulting datasets do not contain independent animal-level biological replicates. Cell-type proportions, module-score distributions, and gene-expression distributions therefore represent descriptive patterns within the pooled datasets and cannot establish animal-level treatment effects. Individual cells were not considered independent biological replicates for treatment-level statistical inference, and sample-aware or animal-level pseudobulk analyses could not be performed because individual animal identities were not retained after pooling. Independent biological replicates will therefore be required to statistically validate these treatment-associated transcriptional and cellular patterns.

Collectively, these findings support CNP-miR146a as a promising therapeutic strategy for diabetic wound repair associated with NRF2-related, ferroptosis-protective myeloid transcriptional programs, endothelial regenerative programs, and predicted immune–vascular communication. The treatment-associated transcriptional changes observed across myeloid and endothelial populations suggest that CNP-miR146a may influence multiple components of the diabetic wound microenvironment. However, the present transcriptomic and computational findings do not establish restoration of myeloid ferroptosis resistance as the principal mechanism driving immune–vascular regeneration. Direct functional and genetic studies will be required to determine whether modulation of myeloid ferroptosis is causally involved in these regenerative responses. Future studies incorporating cell-specific localization and quantification of intracellular miR-146a, together with miR-146a inhibition or genetic loss-of-function approaches, will also be required to determine cell-specific CNP-miR146a delivery and establish the requirement for miR-146a in the observed therapeutic and transcriptional responses.

## 5. Conclusions

CNP-miR146a treatment is associated with coordinated remodeling of ferroptosis-related, antioxidant, iron-homeostatic, and regenerative transcriptional programs in diabetic wounds. Single-cell transcriptomic analysis identified prominent treatment-associated changes within myeloid cells, including enrichment of NRF2-related and ferroptosis-protective transcriptional programs and a repair-associated myeloid state. These changes occurred alongside enhanced endothelial angiogenesis-associated programs and predicted immune–vascular communication networks. Together, these findings extend previous evidence linking NRF2 and ferroptosis regulation to diabetic wound healing by identifying treatment-associated NRF2-related ferroptosis-protective programs within the myeloid compartment and linking these changes to endothelial regenerative programs and predicted immune–vascular communication following CNP-miR146a treatment. Further functional studies measuring ferroptotic cell death and perturbing NRF2 and selected ligand–receptor pathways will be required to establish their causal roles in the therapeutic response.

## Data Availability

The new scRNA-seq data discussed in this publication will be deposited in NCBI’s Gene Expression Omnibus [57] and will be accessible through GEO Series accession numbers at publication.

## Supplementary Materials

The following supporting information can be downloaded at: https://www.mdpi.com/article/10.3390/pharmaceutics18091116/s1. Figure S1: Early Day 3 cellular composition and ferroptosis-associated transcriptional programs in diabetic wounds. Table S1: Single-cell RNA-sequencing quality-control metrics and cell retention across experimental datasets. Table S2: Gene sets used for transcriptional module-score analyses. Table S3: Robustness of transcriptional module scores to alternative gene-set definitions. Figure S2: Sensitivity analysis of transcriptional module-score definitions. Figure S3: CNP-miR146a treatment is associated with increased expression of representative ferroptosis-protective genes across the wound microenvironment.

## Abbreviations

The following abbreviations are used in this manuscript:

APP	Amyloid Precursor Protein
CDI	1,1′-Carbonyldiimidazole
CellChat	Cell–cell communication analysis algorithm
CNP	Cerium Oxide Nanoparticle
CNP-miR146a	Cerium Oxide Nanoparticle-Conjugated microRNA-146a
CXCL4	C-X-C Motif Chemokine Ligand 4
CXCL12	C-X-C Motif Chemokine Ligand 12
DMEM	Dulbecco’s Modified Eagle Medium
FBS	Fetal Bovine Serum
FDR	False Discovery Rate
FN1	Fibronectin 1
HBSS	Hanks’ Balanced Salt Solution
H_2_O_2_	Hydrogen Peroxide
Het PBS	Heterozygous Phosphate-Buffered Saline-treated Control
Hom PBS	Homozygous Diabetic Phosphate-Buffered Saline-treated Control
Hom T	Homozygous Diabetic CNP-miR146a-treated Group
IACUC	Institutional Animal Care and Use Committee
IL6ST	Interleukin 6 Signal Transducer
IRAK1	Interleukin-1 Receptor-Associated Kinase 1
NF-κB	Nuclear Factor Kappa B
NRF2	Nuclear Factor Erythroid 2-Related Factor 2
OSM	Oncostatin M
OSMR	Oncostatin M Receptor
PBS	Phosphate-Buffered Saline
ROS	Reactive Oxygen Species
scRNA-seq	Single-Cell RNA Sequencing
SPP1	Secreted Phosphoprotein 1
THBS	Thrombospondin
TRAF6	TNF Receptor-Associated Factor 6
UMAP	Uniform Manifold Approximation and Projection
VEGF	Vascular Endothelial Growth Factor
