# Supplementary material for: CNP-miR146a Promotes NRF2-Related Ferroptosis-Protective Myeloid Programs and Immune–Vascular Regeneration in Diabetic Wounds
Source: Pharmaceutics. 2026 Sep 4;18(9):1116. doi: 10.3390/pharmaceutics18091116 (PMC13610329; doi:10.3390/pharmaceutics18091116)
Supplement: Supplementary file 1 [file pharmaceutics-18-01116-s001.zip › Supplementary-tables and figures.pdf]

Supplementary Materials: Figure S1: Early Day 3 cellular composition and ferroptosis-associated transcriptional programs in diabetic wounds. **Table S1:** Single-cell RNA-sequencing quality-control metrics and cell retention across experimental datasets. **Table S2:** Gene sets used for transcriptional module-score analyses. **Table S3:** Robustness of transcriptional module scores to alternative gene-set definitions. **Figure S2:** Sensitivity analysis of transcriptional module-score definitions. **Figure S3:** CNP-miR146a treatment is associated with increased expression of representative ferroptosis-protective genes across the wound microenvironment.

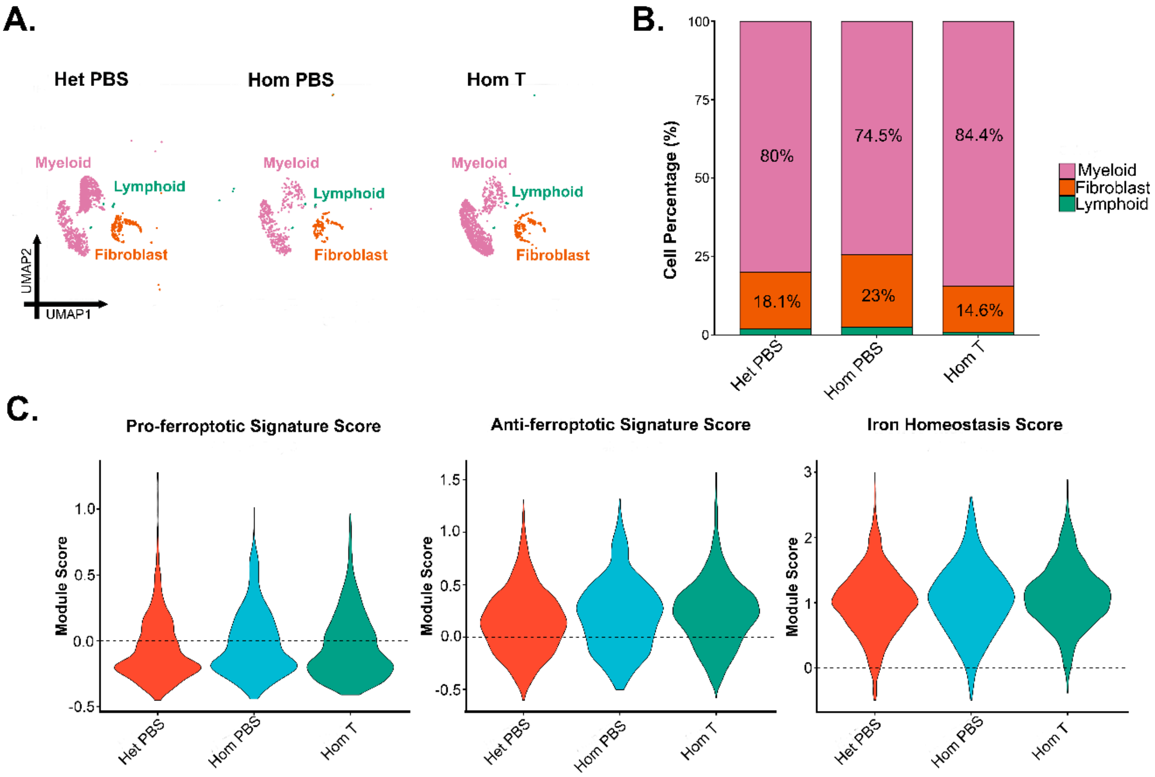

Figure S1. Early Day 3 cellular composition and ferroptosis-associated transcriptional programs in diabetic wounds. **(A)** UMAP visualization of major cell populations in postoperative Day 3 wound tissues, separated by experimental condition (Het PBS, Hom PBS, and Hom T). Major populations included myeloid, fibroblast, and lymphoid cells. **(B)** Relative cell-type composition within each Day 3 experimental group. Myeloid cells represented the predominant population across all conditions, comprising 80.0%, 74.5%, and 84.4% of cells in Het PBS, Hom PBS, and Hom T, respectively. Fibroblasts accounted for 18.1%, 23.0%, and 14.6%, respectively, with lymphoid cells comprising the remaining populations. These percentages describe the cellular composition of the pooled scRNA-seq datasets and were not subjected to animal-level statistical comparisons. **(C)** Distribution of pro-ferroptotic, anti-ferroptosis, and iron-homeostasis module scores across Day 3 experimental groups. Day 3 data are presented as an exploratory early temporal context for the primary Day 7 mechanistic analyses. Module scores represent transcriptional signatures and should not be interpreted as direct measurements of ferroptotic cell death or pathway activity.

Table S1. Single-cell RNA-sequencing quality-control metrics and cell retention across experimental datasets.

Time Point	Dataset	Experimental Group	Before QC	After QC	Retained (%)
Day 3	D3HetPBS	Het PBS	5,275	2,912	55.2
Day 3	D3HomPBS	Hom PBS	1,988	825	41.5
Day 3	D3HomT	Hom T	3,554	1,789	50.3
Day 7	Liechty_DBHT	Het PBS	29,273	19,088	65.2
Day 7	Liechty_DB	Hom PBS	2,920	1,382	47.3
Day 7	D7HomT	Hom T, first run	1,700	580	34.1
Day 7	Liechty_DB_T	Hom T, repeat	13,100	8,447	64.5

Table S2. Gene sets used for transcriptional module-score analyses

Module	Genes included in module-score calculation
Anti-ferroptotic protection	Gpx4, Slc7a11, Slc3a2, Nfe2l2, Hmox1, Nqo1, Gclc, Gclm, Aifm2, Dhodh, Sqstm1
Iron homeostasis	Fth1, Ftl1, Hmox1, Tfrc, Slc40a1, Ncoa4, Steap3, Cp
NRF2-associated antioxidant	Nfe2l2, Hmox1, Nqo1, Gclc, Gclm, Slc7a11, Slc3a2, Fth1, Ftl1, Sqstm1, Txnrd1, Prdx1, Gpx4, Gsr, Sod1, Cat
Pro-ferroptotic	Acsl4, Ptgs2, Alox15, Ncoa4, Tfrc, Steap3

Table S3. Robustness of transcriptional module scores to alternative gene-set definitions.

Module	Definition	Het PBS	Hom PBS	Hom T	Spearman rho	Direction preserved
Anti-ferroptotic protection	Original	0.1190	0.0220	0.1300	0.8120	Yes
	Core Sensitivity	-0.0885	-0.1213	-0.0666		
Iron homeostasis	Original	0.9295	0.5966	0.9219	0.9480	Yes
	Core Sensitivity	0.9156	0.6746	0.9406		
NRF2-associated antioxidant	Original	0.3908	0.2091	0.4038	0.7950	Yes
	Core Sensitivity	0.3078	0.0269	0.2338		
Pro-ferroptotic	Original	0.0282	-0.0366	-0.0050	0.7270	Yes
	Core Sensitivity	0.0450	-0.0600	-0.0199		

Figure S2. Sensitivity analysis of transcriptional module-score definitions.

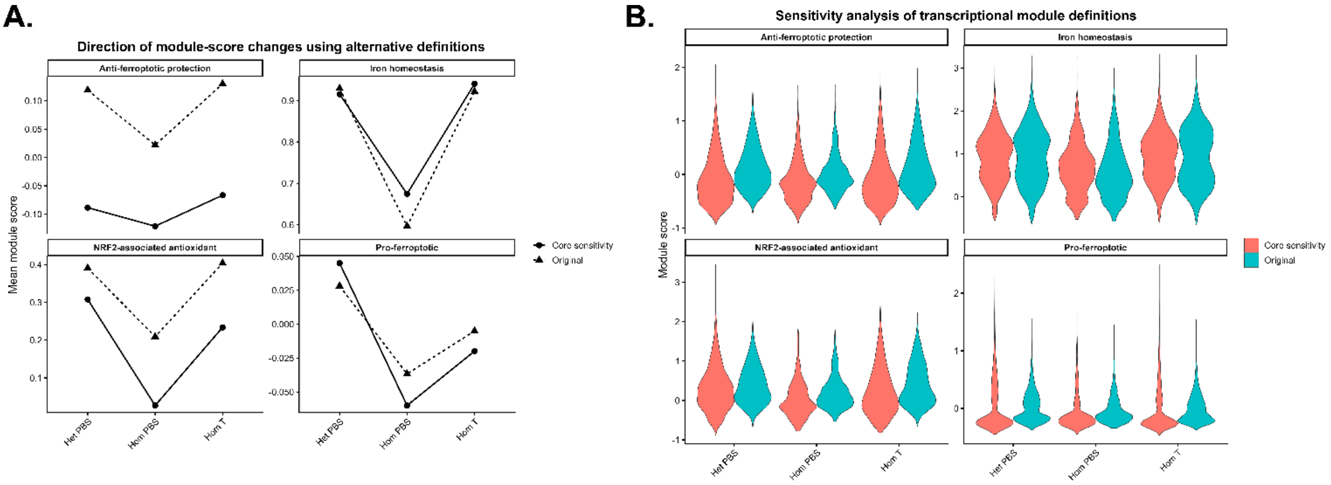

Figure S2. (A) Comparison of mean module scores calculated using the original gene sets used in the primary analysis and reduced core gene sets used for sensitivity analysis across Het PBS, Hom PBS, and Hom T groups. The direction of group-level changes was preserved across alternative gene-set definitions for the anti-ferroptotic protection, iron-homeostasis, NRF2-associated antioxidant, and pro-ferroptotic transcriptional modules. **(B)** Violin plots showing the distributions of module scores calculated using the original and core sensitivity gene sets across experimental groups. Similar group-level patterns across alternative module definitions support the robustness of the observed descriptive transcriptional patterns to gene-set composition. Gene sets used for the original and core sensitivity analyses are provided in Supplementary Table S3.

Figure S3. CNP-miR146a treatment is associated with increased expression of representative ferroptosis-protective genes across the wound microenvironment.

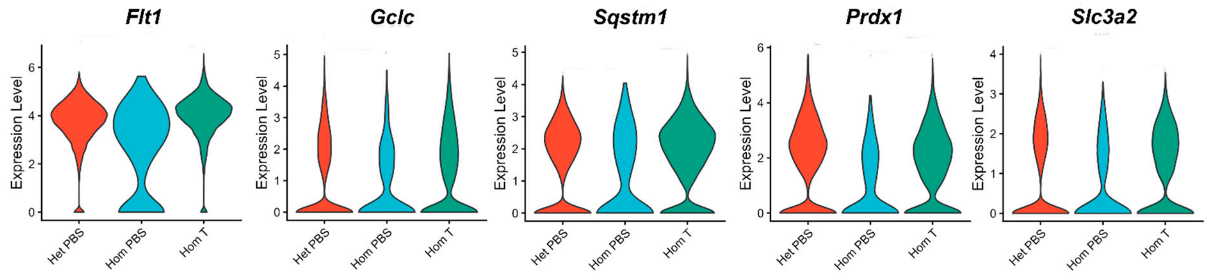

Figure S3. CNP-miR146a is associated with ferroptosis-protective gene expression patterns across the wound microenvironment. Violin plots show normalized expression distributions of representative genes associated with antioxidant defense, glutathione metabolism, and iron homeostasis across all wound cells from the pooled Het PBS, Hom PBS, and Hom T datasets. Genes shown include *Fth1*, *Gclc*, *Sqstm1*, *Prdx1*, and *Slc3a2*. CNP-miR146a-treated diabetic wounds (Hom T) showed higher expression of several ferroptosis-protective genes relative to untreated diabetic wounds (Hom PBS), consistent with the treatment-associated module-score patterns observed in the primary analysis. Expression distributions are presented descriptively because independent animal-level scRNA-seq replicates were not available.
